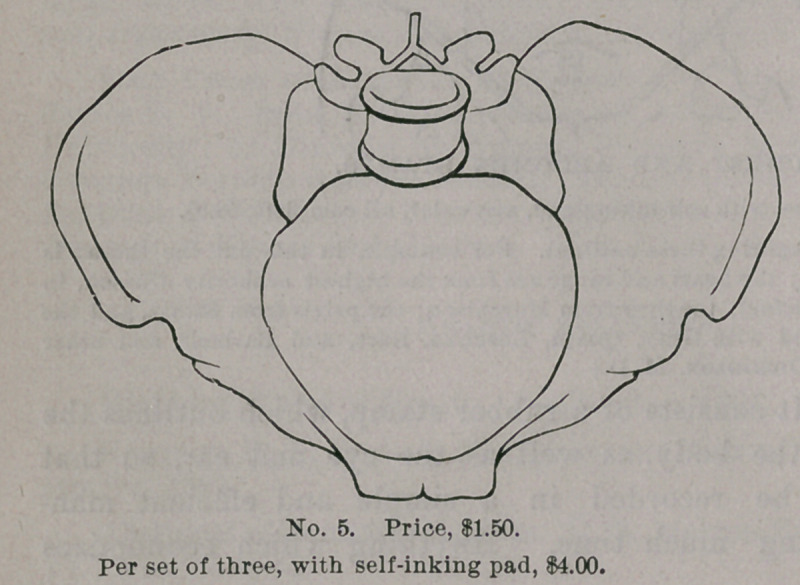# Miscellany

**Published:** 1891-11

**Authors:** 


### Miscellany.

THE DIOS CHEMICAL COMPANY, of St. Louis, has published a fine lithograph of the uterus and its appendages, showing their anatomical relations in colors. This useful drawing will be sent to members of the profession, free, on application. Address the Dios Chemical Company, 914 Locust street, St. Louis, Mo.

### STAMPS FOR RECORDING CASES.

MESSRS. J. C. BARTON & Co., of New York, are the manufacturers of a labor-saving device which should command the attention of every physician. It consists of a rubber stamp, which outlines the several cavities of the body, as well as the eye and ear, so that every case may be recorded in a simple and efficient manner, without taking much time. Anything which economizes

### GYNECOLOGICAL STAMPS.

No. 2. Price, $1.75.

No. 3. Price, $1.25.

time in the present day is to be commended, but this invention does more,— it adds to clearness as well as conciseness, and becomes at once a valuable addition to the equipment of physicians, whether in general or special practice. We subjoin illustrations which explain themselves. These stamps can be purchased of the manufacturers by addressing them at 318 Broadway, New York.

No. 4. Price, $1.25.

No. 5. Price, $1.50.
Per set of three, with self-inking pad, $4.00.

Messrs. J. B. Lippincott & Company announce for early publication the Life of Benjamin Harris Brewster, by Eugene Coleman Savidge, M. D.

Mr. Brewster took an active and important part in many of the most critical and exciting movements in our recent national history. He knew more or less intimately every American celebrity since the times of Webster and Clay, and his biography will be a valuable contribution to the history of the last half century of our national life. It is especially rich in material relative to the Garfield and Arthur administrations, with which he was associated as prosecuting attorney in the famous Star Route trial, and later as Attorney-General. He will also long be remembered because of his brilliant legal and other oratorical efforts, some of the best specimens of which are given in this volume.

---

One of the most valuable acquisitions to medical literature of the year will undoubtedly be the new edition of Prof. Roberts Bartholow's Hypodermic Medication, about to be issued from the press of J. B. Lippincott Company. The rapid progress made in therapeutical science since the last edition appeared, has demanded a thorough revision, in the execution of which Dr. Bartholow has largely re-written the work, describing the various new remedies, and giving the latest results of this method of medication. These changes have increased the work by about two hundred pages, and their importance and value will secure even a higher standing for the work as an authority on this branch of medicine. It will be found indispensable to every physician who would keep abreast with medical progress and discovery.

---

In its November number, the *Cosmopolitan* will publish a series of letters written by General W. T. Sherman to one of his young daughters, between the years 1859 and 1865, and covering most of the important events of the war of secession. These letters present graphic pictures of a great soldier amid some of the stirring scenes in which he was a giant figure, and in them the patriotic spirit of the Federal general is seen to have been most attractively tempered by a strong affection for the Southern people. The fraternal feeling which glows in these letters is in refreshing contrast to the sectional bitterness which characterized the period, and they will constitute an interesting and important contribution to the literature of the war.

Johnson & Johnson, of New York, have published a compilation of recent notes and suggestions from eminent surgeons on the Modern Method of Antiseptic Wound Treatment. This is an octavo pamphlet of forty pages, illustrated, and contains much valuable information for the profession. It will be furnished on application by this well-known house.

---

The second volume of Hermetic Philosophy, by Styx, of the "H. B. of L.," will soon be issued from the press of J. B. Lippincott Company. As in the preceding volume, it includes lessons, general discourses and explanations of Fragments from the schools of Egypt, Chaldea, Greece, Italy, etc., and is a continuation of the line of thought so ably treated in that work.

---

Preserving the Complexion.—A great deal can be done towards having a fine and smooth complexion, by a systematic treatment of rubbing, says *The Ladies' Home Journal.* A fine towel or a bit of red flannel are the best for rubbing, twice a day, or four times, if rapid results are to accrue. By degrees — as the skin gains tone and elasticity from having thrown off the waste matter in its ducts that kept it clogged, sickly, and flabby — the friction can increase in energy. The skin becomes not tougher, but more resistant. If the rubbing is too hard at first, however, it is liable to produce redness and pimples. Even slight friction will do this at times on an unaccustomed skin. But the treatment should be persevered in nevertheless, and the skin soon becomes extraordinarily fine and smooth.

## Figures and Tables

**No. 1. f1:**
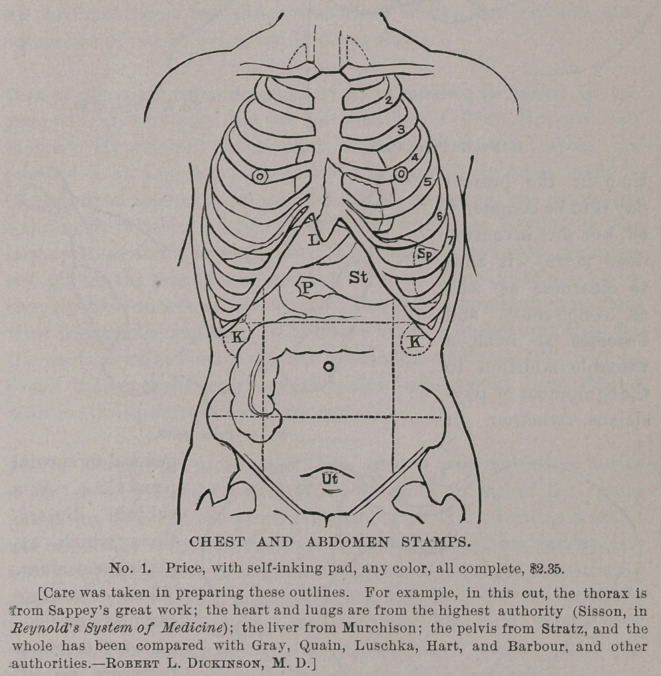


**No. 2. f2:**
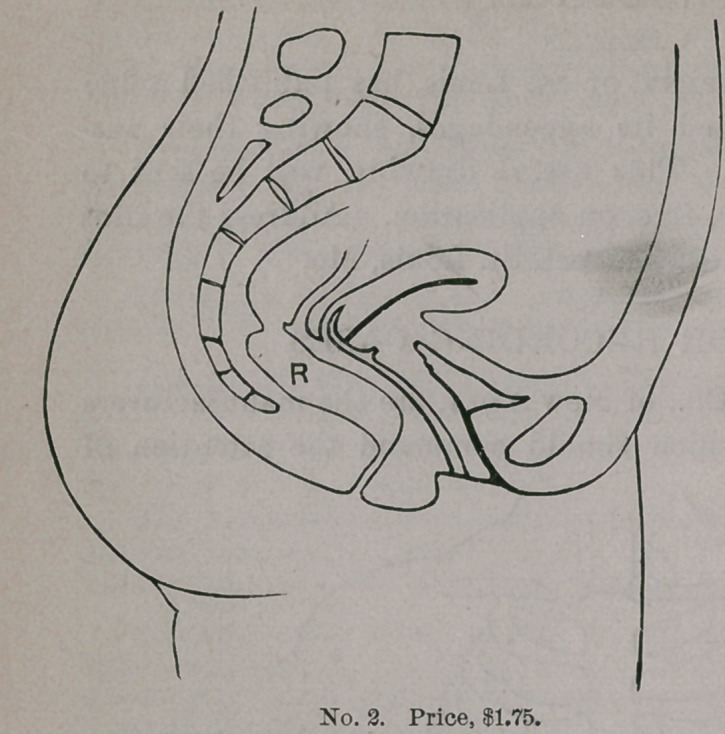


**No. 3. f3:**
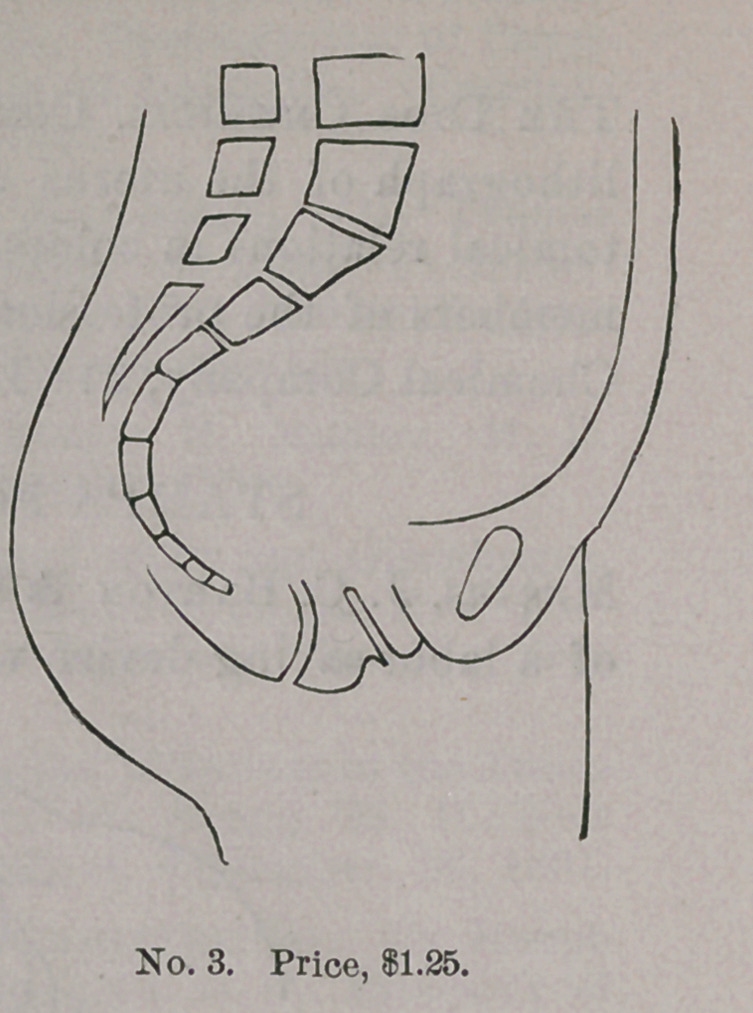


**No. 4. f4:**
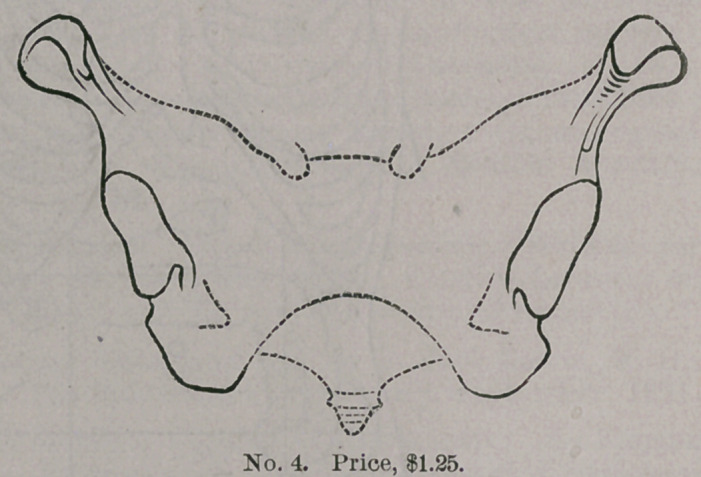


**No. 5. f5:**